# Switching of the Microglial Activation Phenotype Is a Possible Treatment for Depression Disorder

**DOI:** 10.3389/fncel.2018.00306

**Published:** 2018-10-16

**Authors:** Lijuan Zhang, Jinqiang Zhang, Zili You

**Affiliations:** Center for Informational Biology, School of Life Science and Technology, University of Electronic Science and Technology of China, Chengdu, China

**Keywords:** major depressive disorder, inflammation, microglia, neuroprotection, neurogenesis

## Abstract

Major depressive disorder (MDD) is a common emotional cognitive disorder that seriously affects people’s physical and mental health and their quality of life. Due to its clinical and etiological heterogeneity, the molecular mechanisms underpinning MDD are complex and they are not fully understood. In addition, the effects of traditional drug therapy are not ideal. However, postmortem and animal studies have shown that overactivated microglia can inhibit neurogenesis in the hippocampus and induce depressive-like behaviors. Nonetheless, the molecular mechanisms by which microglia regulate nerve regeneration and determine depressive-like behaviors remain unclear. As the immune cells of the central nervous system (CNS), microglia could influence neurogenesis through the M1 and M2 subtypes, and these may promote depressive-like behaviors. Microglia may be divided into four main states or phenotypes. Under stress, microglial cells are induced into the M1 type, releasing inflammatory factors and causing neuroinflammatory responses. After the inflammation fades away, microglia shift into the alternative activated M2 phenotypes that play a role in neuroprotection. These activated M2 subtypes consist of M2a, M2b and M2c and their functions are different in the CNS. In this article, we mainly introduce the relationship between microglia and MDD. Importantly, this article elucidates a plausible mechanism by which microglia regulate inflammation and neurogenesis in ameliorating MDD. This could provide a reliable basis for the treatment of MDD in the future.

## Introduction

Major depressive disorder (MDD) is a common neuropsychiatric disorder with multiple contributing factors—both genetic and environmental—that now affects approximately 350 million people worldwide (Jo et al., 2015). In the past decades, many studies have focused on the monoamine hypothesis. The clinical use of tricyclic antidepressants (TCAs) or serotonin-selective reuptake inhibitors (SSRIs) is targeted at specific neurons and ignores the microenvironment of neurogenesis. Recently, there was a major breakthrough in our understanding of the mechanistic basis for MDD: “inflammatory hypothesis” (Dowlati et al., 2010). Cytokines are pleiotropic molecules with key roles in inflammatory responses and neuroinflammation is important not only in inflammatory responses but also in neurogenesis. Patients with MDD exhibit increased peripheral blood inflammatory biomarkers, including several inflammatory cytokines, such as interleukin (IL)-1β, tumor necrosis factor (TNF)-α and IL-6 (Kim et al., 2007; Dowlati et al., 2010). Proinflammatory cytokines are closely associated with neurogenesis, in that proinflammatory receptors are highly aggregated in hippocampal regions with cognitive functions. For example, IL-1 receptors (IL-1Rs) are distributed in the central nervous system (CNS) of the human, mouse and rat, and they are modulated by various opposing factors including glucocorticoids and anti-inflammatory cytokines to ameliorate sickness behaviors (Parnet et al., 2002). Various studies suggest that proinflammatory cytokines exert harmful effects on cell survival and decrease neurite outgrowth and neural lineage commitment, and proinflammatory cytokines (MIP2, IL-1β, INOS and TNF-α) lead to microglial activation that reduce hippocampal neurogenesis (Monje et al., 2003; Farrell et al., 2016; Wang J. et al., 2018).

Microglial cells are the brain’s immune cells in the CNS. While other major cell populations in the CNS share a neuroepithelial origin, microglia derive from myeloid progenitors, being more closely related to peripheral macrophages than to neurons, neighboring astrocytes, or oligodendrocytes (Ginhoux et al., 2010). They play a role in phagocytes, recognizing and scavenging dead cells and pathogens (Ajami et al., 2007; Casano and Peri, 2015). The presence of neurotransmitter receptors in microglia illustrates their functional connection to neurons and this receptor activation could cause microglial cells to perform different activation phenotypes (Pocock and Kettenmann, 2007). They are involved in various neural activities and immunological functions. Under normal physiological conditions, microglia remain in the resting phenotype involved in neuronal activities such as synaptogenesis, neurogenesis and the release of neurotrophic factors (Bilbo and Schwarz, 2009; Ferrini and De Koninck, 2013; Sato, 2015). However, when the brain is injured and the homeostasis of the microenvironment is disturbed, microglial cells shift into active phenotypes that can secrete proinflammatory cytokines, chemokines, and reactive oxidants (Harry and Kraft, 2008; Lehnardt, 2010). Increases in proinflammatory mediators are likely to damage normal tissues, as the uncontrolled and sustained inflammatory alterations have detrimental effects and further exacerbate the neuronal injury, thus increasing the susceptibility to MDD.

Microglia are characterized by strong plasticity and a diversified morphology and are capable of influencing complex moods, synaptic plasticity, neurogenesis, and memory; hence, some types of depression could be considered as microglial disease (i.e., microgliopathy; Yirmiya et al., 2015). Therefore, analyzing microglia morphology would be a good approach to better understanding the pathogenesis of depression. Here, we first review the potential phenotypic transformation mechanism of microglia, and then describe the impact of their different activation types on neurogenesis as related to depression. Next, we proceed to highlight the interplay between inflammation–microglia and neurogenesis–depression. Lastly, we explain how the key factors impact microglial activation in chronic neurological disorders. Understanding how microglia respond to different stimulators will thus have important implications for controlling the reactivity of these cells in MDD, as well as for treating more chronic neurodegenerative diseases.

## Microglia, Neuroinflammation and MDD

### MDD and Inflammatory Factors

MDD is a serious disease that globally affects many people often accompanied by abnormal behavior, anorexia, lethargy, weight loss, severe feelings of guilt and more sleep (Capuron et al., 2009). Because of its clinical and etiological heterogeneity, the molecular mechanisms underpinning depression are complex and not clearly understood. Inflammatory mediators have been proposed as causal links to MDD. According to some cytokine profile, microglial activation and inflammation are also increased in the brains of MDD patients.

**Figure 1:**
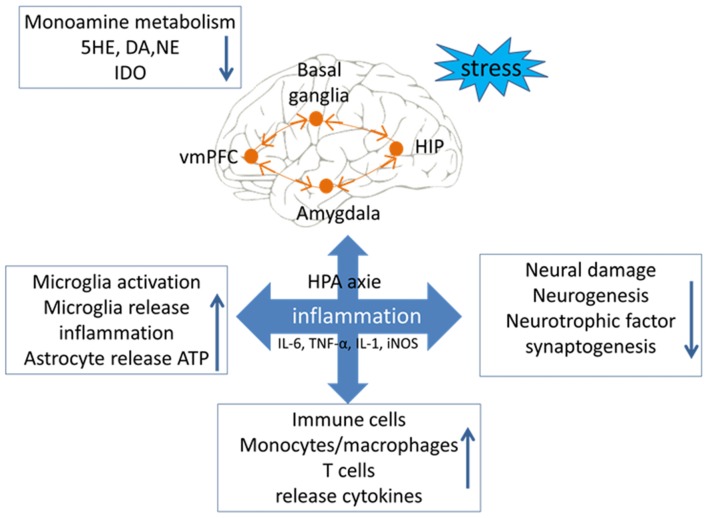
Relationship between inflammation and major depressive disorder (MDD). Stress is an important factor in the occurrence of depression symptoms. There are four main occurrence mechanisms of MDD: (1) stress could lead to disorderly release of neurotransmitters, and then cause an imbalance in the level of neuroinflammatory factors; (2) stress could lead to abnormal intestinal flora and increase the levels of inflammatory factors from the peripheral nervous system and the central nervous system (CNS); (3) stress could lead to excessive activation of microglial cells, causing them to release toxic substances to disrupt the balance of inflammation and anti-inflammation; and (4) stress could lead the immune macrophage to release inflammatory cytokines, which indirectly cause neurological circuit disorders.

Bacterial endotoxin lipopolysaccharide (LPS), a potent activator of proinflammatory cytokines, was found to induce depressive-like behaviors (Medeiros et al., 2015). The sickness-related psychopathological symptoms during infection and inflammation are mediated by increasing multiple proinflammatory cytokines, namely IL-1ß, IL-6, TNF-α and IFN-γ. Intriguingly, an imbalance among them was detected in the serum of patients with MDD (Young et al., 2014; Mao et al., 2018). Some antidepressants have shown strong anti-inflammatory response, for example, selective serotonin and serotonin norepinephrine reuptake inhibitors (SSRI and SNRI, respectively) are the first choice pharmacological options for treating MDD. The transcription of TNF, IL11 and IL6 revealed significant expression differences at baseline and after escitalopram (SSRI antidepressant) treatment in depressed patients (Powell et al., 2013). Yet, it is increasingly apparent that these drugs also exert effects on inhibiting microglial activation (Tynan et al., 2012). The mechanism by which inflammation causes depressive-like behaviors likely involves one or more inflammatory molecules, such as C-reactive protein (CRP) or prostaglandin E2 (PGE2) and the hypothalamus–pituitary–adrenal (HPA) axis (Figure 1).

These observations suggest an interesting connection between microglial inflammatory responses and MDD.

### Microglia Regulate Neuroinflammation in CNS

Although the level of inflammation in the brain is approximately as that in the peripheral areas, our work has shown that peripheral inflammation does not contribute to neuroinflammation in the CNS (You et al., 2011). The peripheral system is separated from the CNS by the blood–brain barrier (BBB), and although most cytokines spans have greater than 15 kDa, they cannot cross the BBB. Although the BBB’s permeability could change under pathological conditions, this condition is ephemeral and dose not last long (Henry et al., 2009). In contrast, MDD is a chronic and persistent psychological disorder. Hence, such permeability changes in the BBB seem insufficient to explain depression which is regulated by specific cells in the brain.

Microglia, the innate immune cells that settle in the brain, account for approximately 5%–20% of the total number of adult glial cells (Polazzi and Monti, 2010; von Bartheld et al., 2016). Microglial cells are bone marrow cells derived from the embryonic yolk sac, which migrate to the brain during early development, and they maintain their abundance in the brain by local self-renewal (Kierdorf et al., 2013; Hoeffel et al., 2015). An interruption in CNS homeostasis could induce a cascade of conserved adaptive responses in microglia. They have retractable branches to search and scan the brain for damage and infection. When parts of the brain are found to be damaged or infected, microglial cells trigger an alarm and a signaling cascade by secreting inflammatory signals (Norden et al., 2015). As more microglia receive these signals, more are recruited to the site of the injury, where they secrete more anti-inflammatory cytokines to resolve the inflammation and also secrete neurotrophic factors to repair the damage (Verney et al., 2010). Since microglia are characterized by diversity and plasticity, it becomes very meaningful to study the effect of differently activated microglia on MDD.

### Microglial Activation and MDD

Microglial activities were recently linked to MDD’s pathological conditions; they have a detrimental effect on neurogenesis by causing neuroinflammation and exacerbating depression (Singhal and Baune, 2017). For example, some models of chronic stresses—such as chronic unpredictable stress, chronic restraint stress, and chronic social defeat stress—can trigger a loss in the number of endogenous hippocampal microglia and cause hippocampal microglial activation (Tong et al., 2017; Wang Y. L. et al., 2018). Many animal studies have shown that changes in microglia structure and function are associated with depressive-like behaviors (Franklin et al., 2018; Wang Y. L. et al., 2018; Wohleb et al., 2018).

Activated microglia have three prominent features: great numbers, an enlarged cell body, and fewer branches. The differently activated phenotypes of microglia are likewise found in the brains of a patient or mouse with depressive-like behaviors (Wang Y. L. et al., 2018). Microglial activation also occurs in MDD patients. Interestingly, autopsies of the anterior cingulate cortex in patients with MDD revealed an activation of microglia and change of inflammation (Steiner et al., 2011), and more activated microglia were found in the ventral prefrontal white matter of patients who had committed suicide after depression (Torres-Platas et al., 2014). In other work, activated microglia appeared in the dorsolateral prefrontal cortex, anterior cingulate cortex and hippocampus of patients with schizophrenia and depression (Steiner et al., 2008, 2011). The increased IBA1 gene expression measured in the white matter of depressed suicides may indicate further evidence for increased microglial priming, as this protein is expressed more highly in the “resting” stage of microglial cells (Imai and Kohsaka, 2002). In summary, together these studies suggest that microglia activation may be considered as an important marker of MDD.

### Activated Phenotypes of Microglia

**Figure 2:**
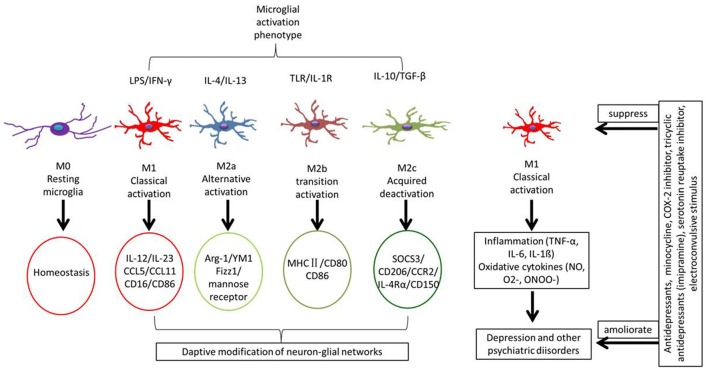
Different microglial phenotypes are related with MDD. Some stimuli induce different types of microglia involving in different functions. Microglial phenotypes are divided into five main states: resting microglia (M0) is mainly to maintain a steady state of the brain’s environment under physiological state; classical activated microglia (M1) is with neurotoxic properties and can release inflammatory cytokines; alternative activated microglia (M2a) is involved in repair and regeneration; transitional activated microglia (M2b) is related to immune regulation; and acquired deactivated microglia (M2c) participate in neuroprotection and release some anti-inflammatory cytokines. Under chronic stress, microglial cells are induced to form the M1 type, releasing inflammatory factors that cause neuroinflammatory reactions.

Due to their heterogeneity, microglia may be induced into several activation phenotypes to detect pathogenic substances and eliminate cell debris, and they can also contribute to nerve regeneration and tissue reconstruction (Li and Barres, 2018). Microglial phenotypes are divided into M1 (Li et al., 2014) and M2 (Almolda et al., 2015), with the latter divided into M2a, M2b and M2c (Franco and Fernández-Suárez, 2015). M1 and M2 microglia can transform each other depending on their activation pathway (Figure 2). Under the action of the IFN-γ cytokine derived from helper T cells (Th1), the resting microglia may turn into the M1 subtype induced by IFN-γ via the classic activation pathway (Prajeeth et al., 2014). Or, conversely, the cytokines IL-4 and IL-13 derived from Type 2 helper T cells (Th2) can induce the transformation of microglia cells into the M2 phenotype through the alternative activation pathway (Ghosh et al., 2016). Crucial participants in this process are interferon regulatory factors (IRFs; Hayakawa et al., 2014), nuclear factor (NF) kappa B (Zhang F. et al., 2017), activator protein 1 (AP1; Wang Y. et al., 2018), and peroxidase (pod) body growth-activated receptor (PPAR)-γ (Pan et al., 2015), which interact with each other to determine the microglial phenotype. The different microglial phenotypes play an important role in regulating the occurrence, development and cessation of inflammatory diseases. Based on the body of evidences, the M1/M2 polarization of microglia contributes significantly to how the production of neuroinflammation is governed in the CNS (Figure 1).

In some chronic neurological diseases, microglial cells become activated and gradually change morphology and function. Microglia could enable the sustained release of proinflammatory mediators leading to the development of diverse neurodegenerative disorders via activating some innate immune signaling pathways, such as the NOD-, LRR- and pyrin domain-containing 3 (NLRP3) inflammasome and so on (de Rivero Vaccari et al., 2009; Adamczak et al., 2014; Heneka et al., 2014). We next argue that the mechanism of microglial activation is associated with MDD.

### The Mechanism of Microglia Activation Associated With MDD

**Figure 3:**
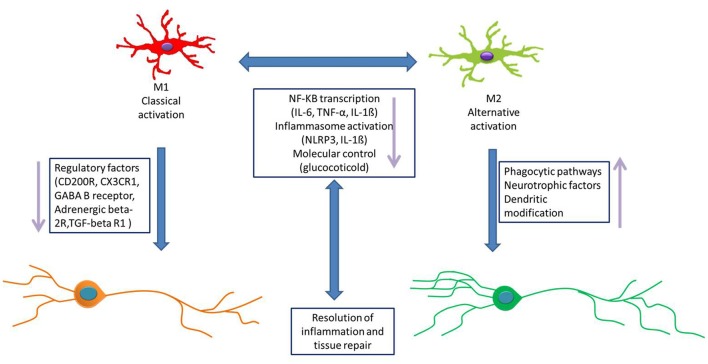
Microglial activation pathway. The activation of microglial cells is affected by many factors. The possible pathways of microglial activation as follows: (1) microglial cells respond to stress through releasing some inflammation, and inflammatory cytokines disrupt the neuronal network, causing MDD. (2) The hypothalamus–pituitary–adrenal (HPA) axis is involved in activated microglia. Intestinal flora could cause changes in the levels of inflammatory factors, which in turn affect the inflammation balance in the CNS and then activate microglia. (3) IL-1ß released from inflammasomes activates microglial cells. Inflammasomes release more inflammatory cytokines, which are amplified by cascade and then activate microglia. (4) Glucocorticoids induce microglia activation. Glucocorticoid-activated microglia respond to a range of immune challenges, including injury, trauma, or infection. (5) The norepinephrine system is also thought to be a stressor of activated microglia. (6) Physiological stress may also trigger the microglia activation directly, by releasing damage-associated molecular patterns (DAMPs) in the brain. (7) Some neurons could directly cause the microglial activation via CX3C receptor 1 (CX3CR1), CD200 and some cytokines.

In clinical depression, how microglial activation occurs is a widely controversial topic that includes several possible mechanisms (Figure 3).

(1) Microglial cells respond to stress through neuroinflammation. This “cytokine hypothesis of depression” proposes that inflammatory cytokines play a key role in the crosswalk of the neurochemical, neuroendocrine, and neurotrophic systems of depressive disorder. It is supported by the evidence that the administration of proinflammatory cytokines in both plasma and CSF have been found to influence the progression and severity of MDD in different populations (Young et al., 2014). Some central and peripheral conditions are closely correlated with the increased incidence of MDD, including stroke, stress, and multiple sclerosis (Anisman and Hayley, 2012). Conservative pathogen- and risk-associated molecular patterns are respectively responsible for the infectious or non-infectious inflammatory microglial response, and microglia are activated by inflammatory factors through humoral and neural pathways. Normally, the interaction between peripheral immune cells and microglia are regulated by the choroid plexus and the BBB. Under pathological conditions (such as viral infection, stroke and trauma), the BBB is weakened and immune cells infiltrate into the brain from the outside. Some damage signals directly stimulate microglia to become activated via their binding to specific receptors, causing them to secrete more inflammatory factors, which thus exacerbate the depression symptoms (de Pablos et al., 2014).

(2) The enteric-brain axis is a system involved in the activation of microglia. In depressed patients, antidepressant treatment has been associated with the dysregulation of the HPA axis (Khemissi et al., 2014). Antidepressant effects of SSRI treatment has been found to alleviate HPA axis dysregulation (Young et al., 2004). Additionally, antidepressant treatment resistance is associated with HPA axis dysfunction. However, the underlying mechanisms are poorly understood (Khemissi et al., 2014). A new article points that the P2X7 receptor antagonist reverses microglial activation and neuroendocrine dysregulation of an unpredictable chronic mild stress model in mice (Farooq et al., 2018). Intestinal flora are controlled under normal physiological conditions and can affect the structure, function, and migration of immune cells. Some studies have shown that gut flora disorders can lead to neurological, hormonal and behavioral changes. For example, hyperactivity of the HPA axis has been associated with higher rates of MDD relapse and chronicity. Moreover, the HPA axis changes tend to improve upon resolution of the depressive syndrome (Juruena, 2014). Depression symptoms were associated with greater cortisol levels and a more prolonged activation of the HPA axis and with an impaired from psychosocial stressors (Lopez-Duran et al., 2015). Under certain situations, stress can increases gut permeability, allowing the gut flora or their metabolites to cross the intestinal mucosa and stimulate the immune-brain system, eventually leading to the activation of immune cells (Feng et al., 2018), resulting in microglia activation that is accompanied by depressive-like behaviors (Depino, 2015).

(3) NLRP3 inflammasome activates microglial cells. Inflammatory processes have been implicated in both acute and chronic stress conditions. The molecular steps leading to IL-1β maturation and a caspase-activating complex take place in an intracellular complex termed the inflammasome (Martinon et al., 2002). NLRP3 (Nucleotide binding and oligomerization domain-like receptor family inflammasome) is a multiprotein complex consisting of NLRP3, pro-caspase-1, and the apoptosis-associated domain (CARD; ASC). There is emerging evidence in animal models that sustained inflammatory responses involving microglia and astrocytes activation contribute to disease progression (Glass et al., 2010). Microglia are the main cell type in the brain responsible for IL-1β and IL-18 secretion. in response to classical inflammasome stimuli (Gustin et al., 2015; Ślusarczyk et al., 2018). Thus microglia-dependent inflammasome activation plays a significant role in the brain and especially in neuroinflammatory condition.

(4) Glucocorticoids induce microglia activation. Most people with MDD showed elevated levels of inflammatory biomarkers. The HPA axis is a major endocrine system that regulates inflammatory responses. Although glucocorticoids are generally anti-inflammatory, they can promote inflammation under certain situations, especially when the brain homeostasis has been disrupted; in this case, glucocorticoids activated microglia respond to a range of immune challenges, including injury, trauma, or infection (Espinosa-Oliva et al., 2011). Glucocorticoid can induce microglia to express the leucine zipper and FK506 binding protein gene 51- itself, mediated by glucocorticoids; hence, these proteins aggravate the stress reaction and depression symptoms. Treatment with corticosterone inhibitor can increase microglial numbers, which further inhibit corticosterone production to eliminate depression symptoms (Nakatani et al., 2012). These results confirm that glucocorticoid can cause the activation of microglia.

(5) The norepinephrine system is also thought to be a stressor of activated microglia. Both social and psychological stress cause the release of norepinephrine, and these signals are released by various immune cells, including microglia. Primary microglia expressed beta(2) adrenergic receptor (AR) and norepinephrine and isoproterenol upregulated the expression of receptor mFPR2, a mouse homolog of human formyl peptide receptor FPR2 that activated beta(2) AR in microglia. Furthermore, the activation of beta(2) AR on microglia induced the expression of an insulin-degrading enzyme and increased the degradation of Aβ42 (Kong et al., 2010). In this way, norepinephrine evidently functions as a link between the neurons and microglia to orchestrate the host response to stress.

(6) Physiological stress may also trigger microglia activation directly by releasing damage-associated molecular patterns (DAMPs) in the brain. The DAMP acts on the toll-like receptor 4 (TLR4) receptor of microglia, producing dangerous signals that are further passed on and cause changes in microglia; specifically, acute stress induces the production of high mobility group box-1 protein (HMGB1) in the DAMP, which enables the microglia to secrete proinflammatory factors, leading to upregulate the gene expression of microglial matrix metal proteins (Kigerl et al., 2018). Stress can also change the expression level of 70 kilodalton heat shock proteins (HSP70) and affect the binding of its molecular partners BAG1, HSP70, and TLR4 receptors, all of which are related to the onset and prevalence of depression (Song et al., 2001).

(7) The activity of neurons causes the activation of microglia. Microglia are involved in synaptic pruning both in development and in the mature CNS. It is now known that, under certain conditions, microglia may adopt a proneurogenic phenotype, which involves the expression of neurotrophins and anti-inflammatory cytokines, such as insulin-like growth factor 1 (IGF-1), brain-derived neurotrophic factor (BDNF), and IL-4 (Ribeiro Xavier et al., 2015; Chen and Trapp, 2016). The physiological functions of microglia are important for maintaining neuronal integrity, network functioning, and neurogenesis in the brain. Stress usually leads to increased neuron activity, and microglial cells may be activated by strong neuronal signals while monitoring environmental changes. Given that the surfaces of microglia have numerous receptors, they can bind to many stress-related neurotransmitters, including glutamate, norepinephrine and serotonin. When faced with stress, threat, anxiety, or pain, we find that many microglial cells are activated and near neurons in the gray matter area of the brain (Hellwig et al., 2016). In addition, chronic mild stress is closely related to the activated microglia and neurons, and pharmacological inhibition of NMDA receptors can inhibit the activation of microglia (Wendt et al., 2016). Clearly then, the activity of neurons interacts with the activation of microglia.

## MDD and Neurogenesis

Because of the heterogeneity of MDD, its pathophysiological mechanism and biological basis remain unclear. Decades of research has clearly shown that a variety of neurotransmitters, especially monoamine neurotransmitters and neurotrophic factors, do contribute to MDD (Joca et al., 2015), which has also been linked to glutamate signaling (Cunningham and Watson, 2008). The hypothesis of depression is based on much related research; for example, brain imaging and postmortem studies in MDD patients indicated the apoptosis of mature neurons and a reduced hippocampal volume, and perhaps more interestingly, that the lag time of antidepressant drugs was approximately equivalent to the cycle of neurogenesis (Sahay and Hen, 2008). In addition, stress is a common risk factor for depression, and long-term stressful conditions can inhibit the neurogenesis of nonhuman primates but this is recoverable via antidepressant therapy. In rodents, inhibiting their hippocampal neurogenesis leads to depression, yet antidepressant drugs could promote neurogenesis. TCA-Imipramine, rarely used in neurogenesis studies, has been shown to increase the proliferation and survival of nerve precursor cells (Zhang et al., 2013). In addition, some drugs, although not approved for use in laboratory animals, did show antidepressant effects, such as CRF1 antagonists and V1B antagonists, which increased the proliferation of neural precursor cells (Henn and Vollmayr, 2004). Nondrug therapy that improves depressive-like behavior also has neurogenic effects. A single electroconvulsive stimulus (ECS), similar to electroconvulsive therapy used for severe depression in clinical practice, can significantly increase the number of newborn neurons that survive (Tang et al., 2016). The stimulation of an electric spasm can also restore to a certain extent the neurogenesis damaged by X-ray irradiation and also restore nerve injuries caused by chronic antidepressant treatments (Santarelli et al., 2003). In addition to influencing both spatial learning and memory, neurogenesis in the hippocampus is also associated with stress-induced depression-and anxiety-like behaviors. An enriched environment can promote the proliferation of nerve precursor cells and alleviate depression and anxiety-like symptoms (Wu et al., 2017). Since different types of antidepressant treatments can increase the proliferation and survival of vital neural precursor cells, this demonstrates that neurogenesis has a positive effect for resisting stress and antidepressant injury, thus suggesting that decreased neurogenesis in the adult hippocampus may be the pathological basis of MDD.

## Microglia and Neurogenesis

According to the activation state of microglial cells, they have two potential functions: supporting or damaging neurogenesis in adult brains. The proinflammatory program (termed M1) microglia often release inflammatory mediators that severely result in the injured tissue (Ding et al., 1988), while the anti-inflammatory phenotypes of microglial cells are neuroprotective type in function and promote the survival of new neurons (Gemma and Bachstetter, 2013). In short, the inflammatory phenotypes of microglial cells often impede neurogenesis. Nonetheless, the effects of different microglial phenotypes on hippocampal neurogenesis are complex and slow.

### Resting Microglia and Neurogenesis

In a healthy brain, most microglial cells are in a resting state. The morphology of resting microglia is poly-branched with many fine branches and a smaller cytoplasm. These cells use their fine branches to detect infections and damage in their environment. In the hippocampal area, resting microglia actively participate in adult neurogenesis through the process of phagocytosis (Sierra et al., 2010). Yet some studies have shown that phagocytic newborn neurons do not trigger microglial activation, indicating that incompletely activated microglial cells also have phagocytic functions. These findings suggest that resting microglia can also regulate, in part, neurogenesis (Sierra et al., 2014). Resting microglia could affect neurogenesis by regulating the function of the neural stem cells *in vitro*, as well as the proliferation and differentiation of neurons by releasing neurotrophic factors (Wadhwa et al., 2017). For example, a review article has shown that over time, the proportion of nerve cells in the subependymal tissue of mice were reduced when they were cultured separately from activated microglia (Shigemoto-Mogami et al., 2014). Another way that neurogenesis is affected is by the disfiguring of microglial receptors, such as ADP receptor P2Y1 (Stefani et al., 2018), vacuolar sorting protein 35 (VPS35; Appel et al., 2018), and CX3C receptor 1 (CX3CR1; Reshef et al., 2014). Thus, the body of work to date suggests that microglia can enhance neurogenesis by secreting unknown factors or via directing contact with neurons. Moreover, it is interesting to note that microglial cells extracted from young rats promoted significantly more neurogenesis than did those from old rats, indicating that the influence of microglial activity weakens with age (Boehme et al., 2014). Together, the evidence suggests that microglial cells support neurogenesis when not activated, thus giving us a better understanding of their functional role in the brain.

### Classical Activation of Microglia on Neurogenesis

There is considerable evidence for the classical activation microglia having a negative effect on neurogenesis in the hippocampus. The bacterial endotoxin LPS can be injected into the CNS or whole body to simulate the inflammatory response in the brain, thereby inducing the classic activation of microglia cells (M1). This activation of microglia by LPS was found to decrease adult neurogenesis, specifically by inhibiting the proliferation or the survival of the new cells (Fujioka and Akema, 2010). LPS with TLR4 molecules induced the microglia activation, and the release of proinflammatory factors, namely IL-1ß, TNFα and IL-6 as well as some other inflammatory molecules (Zhang J. et al., 2017), and an LPS treatment reportedly induced the long-term impairment of hippocampal neurogenesis and memory (Valero et al., 2014). In addition, LPS significantly reduced the number of cells expressing the dual adrenal cortical hormone (DCX), proving that the application of LPS could limit the differentiation of new cells into neurons (Valero et al., 2014). In these studies, the survival of newborn neurons was negatively correlated with the number of microglia activated. In other animal experiments, minocycline, a microglia activity inhibitor, selectively prevented the M1 microglia polarization into a proinflammatory state, providing a basis for understanding the pathogeneses of many diseases accompanied by microglial activation (Kobayashi et al., 2013). Generally, a related report demonstrated that neuroinflammation inhibits neurogenesis in the hippocampus by reducing the differentiation and survival of new neurons (Wang and Jin, 2015). Systemic inflammation, induced by an LPS injection, was sufficient to alter inflammatory status and deregulate the ongoing process of neurogenesis in animals and increased the proliferation of microglia/microglial precursor cells (Smith et al., 2014). Because LPS stimulates M1 microglial activation and this decreased neurogenesis, this strongly suggests that microglial phenotypes are associated with neurogenesis (Zhang J. et al., 2017). Interestingly, over time, aging microglial cells may adopt a potent neurotoxic, proinflammatory “primed” (M1) phenotype when challenged with inflammatory or neurotoxic stimuli that hinder the brain’s own restorative potential and inhibit its endogenous neurorepair mechanisms, and microglia interact with neural stem progenitor cells (NSCs). Microglial subtypes are able to regulate NSCs differently; NSCs from the anti-inflammatory microglial subtype (M2) had better survival and increased migration when kept in a conditioned medium (Osman et al., 2017). This suggests that M2 microglial cells likely contribute to neurogenesis.

### Alternative Activation of Microglia on Neurogenesis

The alternative activated microglia (M2) phenotype operates in both neuroprotection and reconstruction of neural networks in the brain. The M2 microglial cells are distinguished by the release anti-inflammatory mediators, such as IL-4, IL-10 and transforming growth factor-(TGF) ß (Almolda et al., 2015; Franco and Fernández-Suárez, 2015). These inflammatory cytokines inhibit the nonimmune cells from releasing proinflammatory factors. M2 microglia consist of three subtypes (M2a, M2b, M2c). M2a contribute to the repair of damaged tissue by expressing anti-inflammatory and neurotrophic factors; M2b constitute the deactivating phenotype and it also expresses anti-inflammatory mediators; M2c is characterized by its phagocytosis function and associated benefits from clearing out cell debris in the brain (Almolda et al., 2015). Moreover, the M2 microglia supernatant could activate the peroxisome proliferator-activated receptor (PPAR)γ signaling pathway to promote neurogenesis and differentiation of NSPCs (Yuan et al., 2017). Newer research is focusing on the effects of herbal medicines on neurogenesis. For example, naringin dihydrochalcone (NDC), a widely used dietary sweetener with strong antioxidant activity, reduced the abundance of activated microglia and inhibited neuroinflammation, which collectively reduced the neuronal damage (Yang et al., 2018). Salvianolic acid B (SalB), with its anti-inflammatory, antioxidant, and neuroprotective effects, significantly lowered the chronic middle stress (CMS)-induced apoptosis and microglia activation in the hippocampus and cortex (Zhang et al., 2016); it also promoted microglial M2-polarization and rescued neurogenesis in stress-exposed mice (Zhang J. et al., 2017). Indeed, there is compelling evidence that anti-inflammatory factors may promote neurogenesis. Positive correlations were found between the serum IFN-γ/IL-4 ratio and the levels of neurotrophic factors and neurogenesis in the hippocampus (Yang et al., 2016). It was recently revealed that microglial cells secreting IL-10 play a supporting role in the differentiation of neurons and the survival of new cells in cell cultures (Qi et al., 2017). Taken together, these results suggest that M2 microglia do promote neurogenesis differentiation.

Microglia can regulate brain circuit connectivity in multiple ways. During embryonic neurogenesis, they regulate the stem cell pool via their secretion of trophic factors and through phagocytosis (Cunningham et al., 2013). Microglia provide trophic support to neurons and endothelial cells, notably by producing BDNF, IGF-1/2 and TGF-ß such that disrupted growth factor production in microglia could interrupt cortical layer formation (Ueno et al., 2013). Microglial cells are inflammatory phenotypes initially associated with brain injury (Toshimitsu et al., 2018). After ischemic injury to the striatum, microglial cells displayed an M2 phenotype. In addition, this injury also caused new cells to accumulate in the subependymal (SVZ) region of the CNS, as many new cells migrated into the lesion site in the striatum. Prominent changes to microglia activation patterns mainly occur in the alternative activation of neuroprotective phenotypes. In the stroke model, microglial cells in the SVZ region modulate the expression of IL-1β, IL-6, TNF-α and IL-10 (Wang Q. et al., 2018), contributing to neurogenesis. This would suggest that the M2 microglia in particular contribute to the differentiation and migration of neurons. For this reason, microglial cells are now recognized as "gate-keepers" of a healthy brain’s microenvironment, where their disrupted functions adversely affect neurovascular integrity, neuronal homeostasis and network connectivity.

## Microglial Phenotype Switching Is Related to the Treatment of Depression

It has become increasingly evident that different internal or external triggers prompt activated microglia to exert their neurotoxicity or neuroprotection. However, which molecules synergistically interact to switch the states of activated microglia and which act in a gene-specific manner to alter later development of an opposing phenotype are not known. Several studies elucidate that *in vitro*, the former microglia phenotype affects the development of the latter phenotype by initially treating with LPS, IL-4, or IL-10, respectively and subsequently, switching the stimulators’ order (Gresa-Arribas et al., 2012). On one hand, applying an IL-10 pretreatment before administering LPS prevented the expression of TNF-α, IL-6 and COX2; on the other hand, treatment with LPS before an IL-10 introduction led to the expression of inflammation. Alternatively, while pretreatment with IL-4 before LPS failed to inhibit CD86, COX2, INOS and CD32, treatment with LPS before IL-4 prevented the loss of CD32 but did not decrease the M1 marker (Chhor et al., 2013). These contribute evidence for microglia displaying a morphological switch under different activators.

Evidence is mounting to suggest that PPARγ can inhibit microglial activation, promote M2 polarization and suppress inflammatory cytokines in inflammation-related diseases. Depending on the stimulus encountered, the activation profile of microglia transforms from classical activated (M1) to alternative activated (M2) cells (Cherry et al., 2014). In the case of multiple sclerosis, glatiramer acetate (GA) is a promising molecule capable of altering the inflammatory environment by recruiting Th2 T cells to the CNS, inducing the production of IL-4, which ameliorates depressive-like behaviors (Miki et al., 2018). Inflammatory signals may then act on the neuronal network via their neurotoxic activities or by directly influencing mood regulation. A number of inflammatory cytokines, such as IL-1β, TNF-α and IFN-γ, are known to inhibit serotonin transporter activity and ameliorate depressive-like behaviors (Janssen et al., 2010). Microglial cells can come into close contact with neuronal synapses and enhance neurogenesis, thus contributing to MDD.

## The Key Factors to Modulate Microglial Phenotype

Immune cells are needed to clear endogenous or exogenous factors and repair tissue damage, and most of them are resident myeloid cells-microglia. The microglial phenotype is significantly shaped due to the exogenous and endogenous factors.

**Figure 4:**
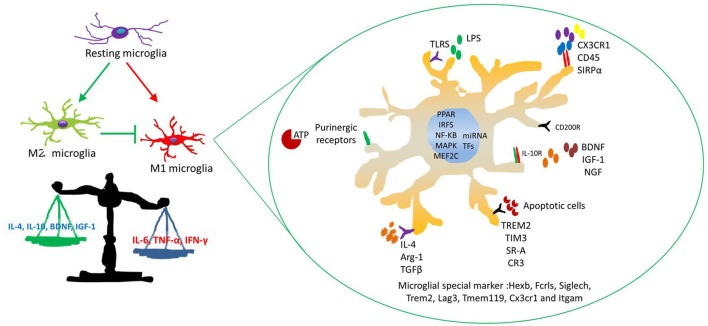
The mechanism of microglial activation. Four major stimuli factors modulate microglial immune activity. These factors are as follows: (a) soluble factors modulate microglial immune responses, such as neurotransmitters and neurotrophic cytokines and so on. (b) Some special receptors are involved in interactions between microglia and neurons. (c) Inflammatory factors and chemokines relate to microglial migration and phagocytosis, such as IL-1ß, IL-10, IL-4 and CCL2, MCP-1 and CXCL1. (d) Some transcription factors (TFs) and microRNAs (miRNAs) affect microglial activation responsed to stress.

Some marker molecules were specially expressed on the microglia in brain regions, such as Cd200r4 and Sirpa (Grabert et al., 2016), while Cx3cr1 was expressed uniformly on the macrophages and microglia (Kim et al., 2011). It is expected that different phenotypes of microglia depend on the communication with neurons. For example, CX3CR1–CX3CL1 signaling may control the microglial phenotype through the release of neuroinflammation; Mef2C and IL-4Rα are highly expressed by microglia in response to TGF-β (Butovsky et al., 2014). Mef2C, as a microglia "off" signal, can alter microglial transcriptome in the presence of type I interferon in aged mouse brain (Deczkowska et al., 2017; Figure 4). Altogether, some complex, spatially diverse cytokine is likely to be involved in controlling microglial activity.

Several endogenous molecule stressors shifting the microglia activation phenotype have been identified, and some studies focus on the key microRNAs (miRNAs) and transcription factors (TFs) in microglia (Guedes et al., 2013; Butovsky et al., 2015). MiRNAs regulate gene expression and are major factors in molecular biology. Several studies indicate that miRNAs participate in the regulation of almost all the cellular processes and the changes in their expression are observed in diseases (Bushati and Cohen, 2007). MiRNAs mediate post-transcription to control cell fate, such as cellular activation and proliferation. An increasing number of studies have revealed that MiRNAs provide a genetic switch to activate target genes via regulating TFs (Arora et al., 2013). For example, CEBPα and PU.1 orchestrate microglial development altogether. CEBPα is being considered as the main regulator in hematopoietic stem cell differentiation (Fazi et al., 2005). MiRNAs are related to microglia activation phenotypes (Ponomarev et al., 2013; Porta et al., 2015); resting microglia cells are characterized by low expression levels of miR-155 and high expression of miR-124; M1 microglia increase the expression levels of miR-155 and decrease the level of miR-124; M2 microglia are distinguished by low expression levels of miR-124 and a high expression of miR-155. MiRNAs are also believed to modulate microglial inflammation stations that contribute to neurogenesis (Freilich et al., 2013; Guedes et al., 2013). Therefore, MiRNA-modulated microglial phenotype therapies could contribute to improve depressive-like behaviors.

## Conclusion

Microglial cells have multiple activation states, namely M1 and M2, that have different effects on modulating neurogenesis in normal and disease conditions. For some, clinical depression is caused by decreasing neurogenesis. Understanding whether the approaches are able to regulate key factors to transform the microglial phenotype for treating MDD is still a great challenge. Much work remains to be done, but it is possible that a deeper understanding of the interplay between different activations of microglia and adult hippocampal neurogenesis may help to develop future strategies to treat MDD.

## Summary

1. The review article elaborately discusses the fact that the activated microglia phenotypes are related to MDD and are helpful for precisely regulating microglia to explore the mechanism of treatment of MDD.
2. The hypothesis of neurogenesis and inflammation, which complement the defects of monoamine hypothesis, gives us a better understanding of the pathogenesis of MDD. Changing the microglial structure and function may link the two hypotheses.
3. The review further elucidates the relationship between inflammation–microglia and neurogenesis–depression symptoms. Such correlations might be of value in the development of a new generation of antidepressants.

## Author Contributions

LZ collected the literature and wrote the manuscript. JZ drew the pictures and revised the article. ZY approved the manuscript and helped in obtaining financial support.

## Conflict of Interest Statement

The authors declare that the research was conducted in the absence of any commercial or financial relationships that could be construed as a potential conflict of interest.

## References

[B1] Adamczak S. E. de Rivero Vaccari J. P. Dale G. Brand F. J. III. Nonner D. Bullock M. R.. (2014). Pyroptotic neuronal cell death mediated by the AIM2 inflammasome. J. Cereb. Blood Flow Metab. 34, 621–629. 10.1038/jcbfm.2013.236 24398937 PMC3982080

[B2] Ajami B. Bennett J. L. Krieger C. Tetzlaff W. Rossi F. M. (2007). Local self-renewal can sustain CNS microglia maintenance and function throughout adult life. Nat. Neurosci. 10, 1538–1543. 10.1038/nn2014 18026097

[B3] Almolda B. de Labra C. Barrera I. Gruart A. Delgado-Garcia J. M. Villacampa N.. (2015). Alterations in microglial phenotype and hippocampal neuronal function in transgenic mice with astrocyte-targeted production of interleukin-10. Brain Behav. Immun. 45, 80–97. 10.1016/j.bbi.2014.10.015 25449577

[B4] Anisman H. Hayley S. (2012). Inflammatory factors contribute to depression and its comorbid conditions. Sci. Signal. 5:pe45. 10.1126/scisignal.2003579 23033537

[B246] Appel J. R. Ye S. Tang F. Sun D. Zhang H. Mei L.. (2018). Increased microglial activity, impaired adult hippocampal neurogenesis, and depressive-like behavior in microglial VPS35-depleted mice. J. Neurosci. 38, 5949–5968. 10.1523/JNEUROSCI.3621-17.2018 29853629 PMC6021995

[B5] Arora S. Rana R. Chhabra A. Jaiswal A. Rani V. (2013). miRNA-transcription factor interactions: a combinatorial regulation of gene expression. Mol. Genet. Genomics 288, 77–87. 10.1007/s00438-013-0734-z 23334784

[B6] Bilbo S. D. Schwarz J. M. (2009). Early-life programming of later-life brain and behavior: a critical role for the immune system. Front. Behav. Neurosci. 3:14. 10.3389/neuro.08.014.2009 19738918 PMC2737431

[B7] Boehme M. Guenther M. Stahr A. Liebmann M. Jaenisch N. Witte O. W.. (2014). Impact of indomethacin on neuroinflammation and hippocampal neurogenesis in aged mice. Neurosci. Lett. 572, 7–12. 10.1016/j.neulet.2014.04.043 24796813

[B10] Bushati N. Cohen S. M. (2007). microRNA functions. Annu. Rev. Cell Dev. Biol. 23, 175–205. 10.1146/annurev.cellbio.23.090506.123406 17506695

[B11] Butovsky O. Jedrychowski M. P. Cialic R. Krasemann S. Murugaiyan G. Fanek Z.. (2015). Targeting miR-155 restores abnormal microglia and attenuates disease in SOD1 mice. Ann. Neurol. 77, 75–99. 10.1002/ana.24304 25381879 PMC4432483

[B12] Butovsky O. Jedrychowski M. P. Moore C. S. Cialic R. Lanser A. J. Gabriely G.. (2014). Identification of a unique TGF-β-dependent molecular and functional signature in microglia. Nat. Neurosci. 17, 131–143. 10.1038/nn.3599 24316888 PMC4066672

[B13] Capuron L. Fornwalt F. B. Knight B. T. Harvey P. D. Ninan P. T. Miller A. H. (2009). Does cytokine-induced depression differ from idiopathic major depression in medically healthy individuals? J. Affect. Disord. 119, 181–185. 10.1016/j.jad.2009.02.017 19269036 PMC2763953

[B14] Casano A. M. Peri F. (2015). Microglia: multitasking specialists of the brain. Dev. Cell 32, 469–477. 10.1016/j.devcel.2015.01.018 25710533

[B16] Chen Z. Trapp B. D. (2016). Microglia and neuroprotection. J. Neurochem. 136, 10–17. 10.1111/jnc.13062 25693054

[B17] Cherry J. D. Olschowka J. A. O’Banion M. K. (2014). Neuroinflammation and M2 microglia: the good, the bad, and the inflamed. J. Neuroinflammation 11:98. 10.1186/1742-2094-11-98 24889886 PMC4060849

[B18] Chhor V. Le Charpentier T. Lebon S. Oré M. V. Celador I. L. Josserand J.. (2013). Characterization of phenotype markers and neuronotoxic potential of polarised primary microglia *in vitro*. Brain Behav. Immun. 32, 70–85. 10.1016/j.bbi.2013.02.005 23454862 PMC3694309

[B19] Cunningham C. L. Martínez-Cerdeño V. Noctor S. C. (2013). Microglia regulate the number of neural precursor cells in the developing cerebral cortex. J. Neurosci. 33, 4216–4233. 10.1523/JNEUROSCI.3441-12.2013 23467340 PMC3711552

[B20] Cunningham K. A. Watson C. S. (2008). Cell cycle regulation, neurogenesis, and depression. Proc. Natl. Acad. Sci. U S A 105, 2259–2260. 10.1073/pnas.0800029105 18272485 PMC2268121

[B22] de Pablos R. M. Herrera A. J. Espinosa-Oliva A. M. Sarmiento M. Muñoz M. F. Machado A.. (2014). Chronic stress enhances microglia activation and exacerbates death of nigral dopaminergic neurons under conditions of inflammation. J. Neuroinflammation 11:34. 10.1186/1742-2094-11-34 24565378 PMC3941799

[B23] de Rivero Vaccari J. P. Lotocki G. Alonso O. F. Bramlett H. M. Dietrich W. D. Keane R. W. (2009). Therapeutic neutralization of the NLRP1 inflammasome reduces the innate immune response and improves histopathology after traumatic brain injury. J. Cereb. Blood Flow Metab. 29, 1251–1261. 10.1038/jcbfm.2009.46 19401709 PMC2846547

[B24] Deczkowska A. Matcovitch-Natan O. Tsitsou-Kampeli A. Ben-Hamo S. Dvir-Szternfeld R. Spinrad A.. (2017). Mef2C restrains microglial inflammatory response and is lost in brain ageing in an IFN-I-dependent manner. Nat. Commun. 8:717. 10.1038/s41467-017-00769-0 28959042 PMC5620041

[B26] Depino A. M. (2015). Early prenatal exposure to LPS results in anxiety- and depression-related behaviors in adulthood. Neuroscience 299, 56–65. 10.1016/j.neuroscience.2015.04.065 25943476

[B27] Ding A. H. Nathan C. F. Stuehr D. J. (1988). Release of reactive nitrogen intermediates and reactive oxygen intermediates from mouse peritoneal macrophages. Comparison of activating cytokines and evidence for independent production. J. Immunol. 141, 2407–2412. 3139757

[B28] Dowlati Y. Herrmann N. Swardfager W. Liu H. Sham L. Reim E. K.. (2010). A meta-analysis of cytokines in major depression. Biol. Psychiatry 67, 446–457. 10.1016/j.biopsych.2009.09.033 20015486

[B29] Espinosa-Oliva A. M. de Pablos R. M. Villarán R. F. Argüelles S. Venero J. L. Machado A.. (2011). Stress is critical for LPS-induced activation of microglia and damage in the rat hippocampus. Neurobiol. Aging 32, 85–102. 10.1016/j.neurobiolaging.2009.01.012 19286276

[B30] Farooq R. K. Tanti A. Ainouche S. Roger S. Belzung C. Camus V. (2018). A P2X7 receptor antagonist reverses behavioural alterations, microglial activation and neuroendocrine dysregulation in an unpredictable chronic mild stress (UCMS) model of depression in mice. Psychoneuroendocrinology 97, 120–130. 10.1016/j.psyneuen.2018.07.016 30015007

[B31] Farrell K. Borazjani A. Damaser M. Kothapalli C. R. (2016). Differential regulation of NSC phenotype and genotype by chronically activated microglia within cocultures. Integr. Biol. Camb. 8, 1145–1157. 10.1039/c6ib00126b 27722366

[B32] Fazi F. Rosa A. Fatica A. Gelmetti V. De Marchis M. L. Nervi C.. (2005). A minicircuitry comprised of microRNA-223 and transcription factors NFI-A and C/EBPα regulates human granulopoiesis. Cell 123, 819–831. 10.1016/j.cell.2005.09.023 16325577

[B300] Feng S. Zou L. Wang H. He R. Liu K. Zhu H. (2018). RhoA/ROCK-2 pathway inhibition and tight junction protein upregulation by catalpol suppresses lipopolysaccaride-induced disruption of blood-brain barrier permeability. Molecules 23:E2371. 10.3390/molecules23092371 30227623 PMC6225311

[B34] Ferrini F. De Koninck Y. (2013). Microglia control neuronal network excitability via BDNF signalling. Neural Plast. 2013:429815. 10.1155/2013/429815 24089642 PMC3780625

[B35] Franco R. Fernández-Suárez D. (2015). Alternatively activated microglia and macrophages in the central nervous system. Prog. Neurobiol. 131, 65–86. 10.1016/j.pneurobio.2015.05.003 26067058

[B36] Franklin T. C. Wohleb E. S. Zhang Y. Fogaça M. Hare B. Duman R. S. (2018). Persistent increase in microglial RAGE contributes to chronic stress-induced priming of depressive-like behavior. Biol. Psychiatry 83, 50–60. 10.1016/j.biopsych.2017.06.034 28882317 PMC6369917

[B37] Freilich R. W. Woodbury M. E. Ikezu T. (2013). Integrated expression profiles of mRNA and miRNA in polarized primary murine microglia. PLoS One 8:e79416. 10.1371/journal.pone.0079416 24244499 PMC3823621

[B39] Fujioka H. Akema T. (2010). Lipopolysaccharide acutely inhibits proliferation of neural precursor cells in the dentate gyrus in adult rats. Brain Res. 1352, 35–42. 10.1016/j.brainres.2010.07.032 20647006

[B40] Gemma C. Bachstetter A. D. (2013). The role of microglia in adult hippocampal neurogenesis. Front. Cell. Neurosci. 7:229. 10.3389/fncel.2013.00229 24319411 PMC3837350

[B41] Ghosh M. Xu Y. Pearse D. D. (2016). Cyclic AMP is a key regulator of M1 to M2a phenotypic conversion of microglia in the presence of Th2 cytokines. J. Neuroinflammation 13:9. 10.1186/s12974-015-0463-9 26757726 PMC4711034

[B42] Ginhoux F. Greter M. Leboeuf M. Nandi S. See P. Gokhan S.. (2010). Fate mapping analysis reveals that adult microglia derive from primitive macrophages. Science 330, 841–845. 10.1126/science.1194637 20966214 PMC3719181

[B501] Glass C. K. Saijo K. Winner B. Marchetto M. C. Gage F. H. (2010). Mechanisms underlying inflammation in neurodegeneration. Cell 140, 918–934. 10.1016/j.cell.2010.02.016 20303880 PMC2873093

[B43] Grabert K. Michoel T. Karavolos M. H. Clohisey S. Baillie J. K. Stevens M. P.. (2016). Microglial brain region-dependent diversity and selective regional sensitivities to aging. Nat. Neurosci. 19, 504–516. 10.1038/nn.4222 26780511 PMC4768346

[B45] Gresa-Arribas N. Vieitez C. Dentesano G. Serratosa J. Saura J. Solà C. (2012). Modelling neuroinflammation *in vitro*: a tool to test the potential neuroprotective effect of anti-inflammatory agents. PLoS One 7:e45227. 10.1371/journal.pone.0045227 23028862 PMC3447933

[B46] Guedes J. Cardoso A. L. Pedroso de Lima M. C. (2013). Involvement of microRNA in microglia-mediated immune response. Clin. Dev. Immunol. 2013:186872. 10.1155/2013/186872 23762086 PMC3676986

[B500] Gustin A. Kirchmeyer M. Koncina E. Felten P. Losciuto S. Heurtaux T.. (2015). NLRP3 inflammasome is expressed and functional in mouse brain microglia but not in astrocytes. PLoS One 10:e0130624. 10.1371/journal.pone.0130624 26091541 PMC4474809

[B47] Harry G. J. Kraft A. D. (2008). Neuroinflammation and microglia: considerations and approaches for neurotoxicity assessment. Expert Opin. Drug Metab. Toxicol. 4, 1265–1277. 10.1517/17425255.4.10.1265 18798697 PMC2658618

[B48] Hayakawa K. Okazaki R. Morioka K. Nakamura K. Tanaka S. Ogata T. (2014). Lipopolysaccharide preconditioning facilitates M2 activation of resident microglia after spinal cord injury. J. Neurosci. Res. 92, 1647–1658. 10.1002/jnr.23448 25044014

[B49] Hellwig S. Brioschi S. Dieni S. Frings L. Masuch A. Blank T.. (2016). Altered microglia morphology and higher resilience to stress-induced depression-like behavior in CX3CR1-deficient mice. Brain Behav. Immun. 55, 126–137. 10.1016/j.bbi.2015.11.008 26576722

[B50] Heneka M. T. Kummer M. P. Latz E. (2014). Innate immune activation in neurodegenerative disease. Nat. Rev. Immunol. 14, 463–477. 10.1038/nri3705 24962261

[B51] Henn F. A. Vollmayr B. (2004). Neurogenesis and depression: etiology or epiphenomenon? Biol. Psychiatry 56, 146–150. 10.1016/j.biopsych.2004.04.011 15271582

[B52] Henry C. J. Huang Y. Wynne A. M. Godbout J. P. (2009). Peripheral lipopolysaccharide (LPS) challenge promotes microglial hyperactivity in aged mice that is associated with exaggerated induction of both pro-inflammatory IL-1β and anti-inflammatory IL-10 cytokines. Brain Behav. Immun. 23, 309–317. 10.1016/j.bbi.2008.09.002 18814846 PMC2692986

[B53] Hoeffel G. Chen J. Lavin Y. Low D. Almeida F. F. See P.. (2015). C-Myb^+^ erythro-myeloid progenitor-derived fetal monocytes give rise to adult tissue-resident macrophages. Immunity 42, 665–678. 10.1016/j.immuni.2015.03.011 25902481 PMC4545768

[B55] Imai Y. Kohsaka S. (2002). Intracellular signaling in M-CSF-induced microglia activation: role of Iba1. Glia 40, 164–174. 10.1002/glia.10149 12379904

[B57] Janssen D. G. Caniato R. N. Verster J. C. Baune B. T. (2010). A psychoneuroimmunological review on cytokines involved in antidepressant treatment response. Hum. Psychopharmacol. 25, 201–215. 10.1002/hup.1103 20373471

[B58] Jo W. K. Zhang Y. Emrich H. M. Dietrich D. E. (2015). Glia in the cytokine-mediated onset of depression: fine tuning the immune response. Front. Cell. Neurosci. 9:268. 10.3389/fncel.2015.00268 26217190 PMC4498101

[B59] Joca S. R. Moreira F. A. Wegener G. (2015). Atypical neurotransmitters and the neurobiology of depression. CNS Neurol. Disord. Drug Targets 14, 1001–1011. 10.2174/1871527314666150909114804 26350337

[B60] Juruena M. F. (2014). Early-life stress and HPA axis trigger recurrent adulthood depression. Epilepsy Behav. 38, 148–159. 10.1016/j.yebeh.2013.10.020 24269030

[B61] Khemissi W. Farooq R. K. Le Guisquet A. M. Sakly M. Belzung C. (2014). Dysregulation of the hypothalamus-pituitary-adrenal axis predicts some aspects of the behavioral response to chronic fluoxetine: association with hippocampal cell proliferation. Front. Behav. Neurosci. 8:340. 10.3389/fnbeh.2014.00340 25324748 PMC4179749

[B62] Kierdorf K. Erny D. Goldmann T. Sander V. Schulz C. Perdiguero E. G.. (2013). Microglia emerge from erythromyeloid precursors via Pu.1- and Irf8-dependent pathways. Nat. Neurosci. 16, 273–280. 10.1038/nn.3318 23334579

[B63] Kigerl K. A. Lai W. Wallace L. M. Yang H. Popovich P. G. (2018). High mobility group box-1 (HMGB1) is increased in injured mouse spinal cord and can elicit neurotoxic inflammation. Brain Behav. Immun. 72, 22–33. 10.1016/j.bbi.2017.11.018 29175543 PMC6681463

[B65] Kim Y. K. Na K. S. Shin K. H. Jung H. Y. Choi S. H. Kim J. B. (2007). Cytokine imbalance in the pathophysiology of major depressive disorder. Prog. Neuropsychopharmacol. Biol. Psychiatry 31, 1044–1053. 10.1016/j.pnpbp.2007.03.004 17433516

[B64] Kim K. W. Vallon-Eberhard A. Zigmond E. Farache J. Shezen E. Shakhar G.. (2011). *In vivo* structure/function and expression analysis of the CX3C chemokine fractalkine. Blood 118, e156–e167. 10.1182/blood-2011-04-348946 21951685 PMC4507037

[B66] Kobayashi K. Imagama S. Ohgomori T. Hirano K. Uchimura K. Sakamoto K.. (2013). Minocycline selectively inhibits M1 polarization of microglia. Cell Death Dis. 4:e525. 10.1038/cddis.2013.54 23470532 PMC3613832

[B67] Kong Y. Ruan L. Qian L. Liu X. Le Y. (2010). Norepinephrine promotes microglia to uptake and degrade amyloid β peptide through upregulation of mouse formyl peptide receptor 2 and induction of insulin-degrading enzyme. J. Neurosci. 30, 11848–11857. 10.1523/JNEUROSCI.2985-10.2010 20810904 PMC6633413

[B68] Lehnardt S. (2010). Innate immunity and neuroinflammation in the CNS: the role of microglia in Toll-like receptor-mediated neuronal injury. Glia 58, 253–263. 10.1002/glia.20928 19705460

[B70] Li Q. Barres B. A. (2018). Microglia and macrophages in brain homeostasis and disease. Nat. Rev. Immunol. 18, 225–242. 10.1038/nri.2017.125 29151590

[B71] Li Z. Ma L. Kulesskaya N. Võikar V. Tian L. (2014). Microglia are polarized to M1 type in high-anxiety inbred mice in response to lipopolysaccharide challenge. Brain Behav. Immun. 38, 237–248. 10.1016/j.bbi.2014.02.008 24561490

[B72] Lopez-Duran N. L. McGinnis E. Kuhlman K. Geiss E. Vargas I. Mayer S. (2015). HPA-axis stress reactivity in youth depression: evidence of impaired regulatory processes in depressed boys. Stress 18, 545–553. 10.3109/10253890.2015.1053455 26115161 PMC5403248

[B503] Martinon F. Burns K. Tschopp J. (2002). The inflammasome: a molecular platform triggering activation of inflammatory caspases and processing of proIL-β. Mol. Cell. 10, 417–426. 10.1016/S1097-2765(02)00599-3 12191486

[B73] Mao R. Zhang C. Chen J. Zhao G. Zhou R. Wang F.. (2018). Different levels of pro- and anti-inflammatory cytokines in patients with unipolar and bipolar depression. J. Affect. Disord. 237, 65–72. 10.1016/j.jad.2018.04.115 29778935

[B74] Medeiros I. U. Ruzza C. Asth L. Guerrini R. Romão P. R. Gavioli E. C.. (2015). Blockade of nociceptin/orphanin FQ receptor signaling reverses LPS-induced depressive-like behavior in mice. Peptides 72, 95–103. 10.1016/j.peptides.2015.05.006 26028163

[B75] Miki A. Honda S. Inoue Y. Yamada Y. Nakamura M. (2018). Foveal depression and related factors in patients with a history of retinopathy of prematurity. Ophthalmologica 240, 106–110. 10.1159/000488368 29742514

[B77] Monje M. L. Toda H. Palmer T. D. (2003). Inflammatory blockade restores adult hippocampal neurogenesis. Science 302, 1760–1765. 10.1126/science.1088417 14615545

[B79] Nakatani Y. Amano T. Tsuji M. Takeda H. (2012). Corticosterone suppresses the proliferation of BV2 microglia cells via glucocorticoid, but not mineralocorticoid receptor. Life Sci. 91, 761–770. 10.1016/j.lfs.2012.08.019 22940619

[B81] Norden D. M. Muccigrosso M. M. Godbout J. P. (2015). Microglial priming and enhanced reactivity to secondary insult in aging and traumatic CNS injury and neurodegenerative disease. Neuropharmacology 96, 29–41. 10.1016/j.neuropharm.2014.10.028 25445485 PMC4430467

[B83] Osman A. M. Rodhe J. Shen X. Dominguez C. A. Joseph B. Blomgren K. (2017). The secretome of microglia regulate neural stem cell function. Neuroscience [Epub ahead of print]. 10.1016/j.neuroscience.2017.10.034 29101080

[B84] Pan J. Jin J. L. Ge H. M. Yin K. L. Chen X. Han L. J.. (2015). Malibatol A regulates microglia M1/M2 polarization in experimental stroke in a PPARγ-dependent manner. J. Neuroinflammation 12:51. 10.1186/s12974-015-0270-3 25889216 PMC4378556

[B86] Parnet P. Kelley K. W. Bluthé R. M. Dantzer R. (2002). Expression and regulation of interleukin-1 receptors in the brain. Role in cytokines-induced sickness behavior. J. Neuroimmunol. 125, 5–14. 10.1016/s0165-5728(02)00022-x 11960635

[B88] Pocock J. M. Kettenmann H. (2007). Neurotransmitter receptors on microglia. Trends Neurosci. 30, 527–535. 10.1016/j.tins.2007.07.007 17904651

[B89] Polazzi E. Monti B. (2010). Microglia and neuroprotection: from *in vitro* studies to therapeutic applications. Prog. Neurobiol. 92, 293–315. 10.1016/j.pneurobio.2010.06.009 20609379

[B90] Ponomarev E. D. Veremeyko T. Weiner H. L. (2013). MicroRNAs are universal regulators of differentiation, activation and polarization of microglia and macrophages in normal and diseased CNS. Glia 61, 91–103. 10.1002/glia.22363 22653784 PMC3434289

[B91] Porta C. Riboldi E. Ippolito A. Sica A. (2015). Molecular and epigenetic basis of macrophage polarized activation. Semin. Immunol. 27, 237–248. 10.1016/j.smim.2015.10.003 26561250

[B92] Powell T. R. Schalkwyk L. C. Heffernan A. L. Breen G. Lawrence T. Price T.. (2013). Tumor necrosis factor and its targets in the inflammatory cytokine pathway are identified as putative transcriptomic biomarkers for escitalopram response. Eur. Neuropsychopharmacol. 23, 1105–1114. 10.1016/j.euroneuro.2012.09.009 23142150

[B93] Prajeeth C. K. Löhr K. Floess S. Zimmermann J. Ulrich R. Gudi V.. (2014). Effector molecules released by Th1 but not Th17 cells drive an M1 response in microglia. Brain Behav. Immun. 37, 248–259. 10.1016/j.bbi.2014.01.001 24412213

[B94] Qi F. Zuo Z. Yang J. Hu S. Yang Y. Yuan Q.. (2017). Combined effect of BCG vaccination and enriched environment promote neurogenesis and spatial cognition via a shift in meningeal macrophage M2 polarization. J. Neuroinflammation 14:32. 10.1186/s12974-017-0808-7 28183352 PMC5301319

[B97] Reshef R. Kreisel T. Beroukhim Kay D. Yirmiya R. (2014). Microglia and their CX3CR1 signaling are involved in hippocampal- but not olfactory bulb-related memory and neurogenesis. Brain Behav. Immun. 41, 239–250. 10.1016/j.bbi.2014.04.009 24933434

[B98] Ribeiro Xavier A. L. Kress B. T. Goldman S. A. Lacerda de Menezes J. R. Nedergaard M. (2015). A distinct population of microglia supports adult neurogenesis in the subventricular zone. J. Neurosci. 35, 11848–11861. 10.1523/JNEUROSCI.1217-15.2015 26311768 PMC4549398

[B100] Sahay A. Hen R. (2008). Hippocampal neurogenesis and depression. Novartis Found. Symp. 289, 152–160; discussion 160–154, 193–155. 10.1002/9780470751251.ch12 18497101

[B101] Santarelli L. Saxe M. Gross C. Surget A. Battaglia F. Dulawa S.. (2003). Requirement of hippocampal neurogenesis for the behavioral effects of antidepressants. Science 301, 805–809. 10.1126/science.1083328 12907793

[B103] Sato K. (2015). Effects of microglia on neurogenesis. Glia 63, 1394–1405. 10.1002/glia.22858 26010551 PMC5032973

[B105] Shigemoto-Mogami Y. Hoshikawa K. Goldman J. E. Sekino Y. Sato K. (2014). Microglia enhance neurogenesis and oligodendrogenesis in the early postnatal subventricular zone. J. Neurosci. 34, 2231–2243. 10.1523/JNEUROSCI.1619-13.2014 24501362 PMC3913870

[B106] Sierra A. Beccari S. Diaz-Aparicio I. Encinas J. M. Comeau S. Tremblay M. E. (2014). Surveillance, phagocytosis, and inflammation: how never-resting microglia influence adult hippocampal neurogenesis. Neural Plast. 2014:610343. 10.1155/2014/610343 24772353 PMC3977558

[B107] Sierra A. Encinas J. M. Deudero J. J. Chancey J. H. Enikolopov G. Overstreet-Wadiche L. S.. (2010). Microglia shape adult hippocampal neurogenesis through apoptosis-coupled phagocytosis. Cell Stem Cell 7, 483–495. 10.1016/j.stem.2010.08.014 20887954 PMC4008496

[B108] Singhal G. Baune B. T. (2017). Microglia: an interface between the loss of neuroplasticity and depression. Front. Cell. Neurosci. 11:270. 10.3389/fncel.2017.00270 28943841 PMC5596091

[B109] Ślusarczyk J. Trojan E. Głombik K. Piotrowska A. Budziszewska B. Kubera M.. (2018). Targeting the NLRP3 inflammasome-related pathways via tianeptine treatment-suppressed microglia polarization to the M1 phenotype in lipopolysaccharide-stimulated cultures. Int. J. Mol. Sci. 19:E1965. 10.3390/ijms19071965 29976873 PMC6073715

[B110] Smith P. L. Hagberg H. Naylor A. S. Mallard C. (2014). Neonatal peripheral immune challenge activates microglia and inhibits neurogenesis in the developing murine hippocampus. Dev. Neurosci. 36, 119–131. 10.1159/000359950 24642725

[B111] Song J. Takeda M. Morimoto R. I. (2001). Bag1-Hsp70 mediates a physiological stress signalling pathway that regulates Raf-1/ERK and cell growth. Nat. Cell Biol. 3, 276–282. 10.1038/35060068 11231577

[B112] Stefani J. Tschesnokowa O. Parrilla M. Robaye B. Boeynaems J. M. Acker-Palmer A.. (2018). Disruption of the microglial ADP receptor P2Y13 enhances adult hippocampal neurogenesis. Front. Cell. Neurosci. 12:134. 10.3389/fncel.2018.00134 29867367 PMC5966569

[B113] Steiner J. Bielau H. Brisch R. Danos P. Ullrich O. Mawrin C.. (2008). Immunological aspects in the neurobiology of suicide: elevated microglial density in schizophrenia and depression is associated with suicide. J. Psychiatr. Res. 42, 151–157. 10.1016/j.jpsychires.2006.10.013 17174336

[B114] Steiner J. Walter M. Gos T. Guillemin G. J. Bernstein H. G. Sarnyai Z.. (2011). Severe depression is associated with increased microglial quinolinic acid in subregions of the anterior cingulate gyrus: evidence for an immune-modulated glutamatergic neurotransmission? J. Neuroinflammation 8:94. 10.1186/1742-2094-8-94 21831269 PMC3177898

[B117] Tang M. M. Lin W. J. Pan Y. Q. Guan X. T. Li Y. C. (2016). Hippocampal neurogenesis dysfunction linked to depressive-like behaviors in a neuroinflammation induced model of depression. Physiol. Behav. 161, 166–173. 10.1016/j.physbeh.2016.04.034 27106565

[B119] Tong L. Gong Y. Wang P. Hu W. Wang J. Chen Z.. (2017). Microglia loss contributes to the development of major depression induced by different types of chronic stresses. Neurochem. Res. 42, 2698–2711. 10.1007/s11064-017-2270-4 28434164

[B120] Torres-Platas S. G. Cruceanu C. Chen G. G. Turecki G. Mechawar N. (2014). Evidence for increased microglial priming and macrophage recruitment in the dorsal anterior cingulate white matter of depressed suicides. Brain Behav. Immun. 42, 50–59. 10.1016/j.bbi.2014.05.007 24858659

[B121] Toshimitsu M. Kamei Y. Ichinose M. Seyama T. Imada S. Iriyama T.. (2018). Atomoxetine, a selective norepinephrine reuptake inhibitor, improves short-term histological outcomes after hypoxic-ischemic brain injury in the neonatal male rat. Int. J. Dev. Neurosci. [Epub ahead of print]. 10.1016/j.ijdevneu.2018.03.011 29608930

[B122] Tynan R. J. Weidenhofer J. Hinwood M. Cairns M. J. Day T. A. Walker F. R. (2012). A comparative examination of the anti-inflammatory effects of SSRI and SNRI antidepressants on LPS stimulated microglia. Brain Behav. Immun. 26, 469–479. 10.1016/j.bbi.2011.12.011 22251606

[B124] Ueno M. Fujita Y. Tanaka T. Nakamura Y. Kikuta J. Ishii M.. (2013). Layer V cortical neurons require microglial support for survival during postnatal development. Nat. Neurosci. 16, 543–551. 10.1038/nn.3358 23525041

[B125] Valero J. Mastrella G. Neiva I. Sanchez S. Malva J. O. (2014). Long-term effects of an acute and systemic administration of LPS on adult neurogenesis and spatial memory. Front. Neurosci. 8:83. 10.3389/fnins.2014.00083 24795557 PMC4001049

[B126] Verney C. Monier A. Fallet-Bianco C. Gressens P. (2010). Early microglial colonization of the human forebrain and possible involvement in periventricular white-matter injury of preterm infants. J. Anat. 217, 436–448. 10.1111/j.1469-7580.2010.01245.x 20557401 PMC2992419

[B127] von Bartheld C. S. Bahney J. Herculano-Houzel S. (2016). The search for true numbers of neurons and glial cells in the human brain: a review of 150 years of cell counting. J. Comp. Neurol. 524, 3865–3895. 10.1002/cne.24040 27187682 PMC5063692

[B128] Wadhwa M. Prabhakar A. Ray K. Roy K. Kumari P. Jha P. K.. (2017). Inhibiting the microglia activation improves the spatial memory and adult neurogenesis in rat hippocampus during 48 h of sleep deprivation. J. Neuroinflammation 14:222. 10.1186/s12974-017-0998-z 29141671 PMC5688670

[B134] Wang Y. L. Han Q. Q. Gong W. Q. Pan D. H. Wang L. Z. Hu W.. (2018). Microglial activation mediates chronic mild stress-induced depressive- and anxiety-like behavior in adult rats. J. Neuroinflammation 15:21. 10.1186/s12974-018-1054-3 29343269 PMC5773028

[B133] Wang Y. Huang Y. Xu Y. Ruan W. Wang H. Zhang Y.. (2018). A dual AMPK/Nrf2 activator reduces brain inflammation after stroke by enhancing microglia M2 polarization. Antioxid. Redox Signal. 28, 141–163. 10.1089/ars.2017.7003 28747068

[B130] Wang B. Jin K. (2015). Current perspectives on the link between neuroinflammation and neurogenesis. Metab. Brain Dis. 30, 355–365. 10.1007/s11011-014-9523-6 24623361

[B131] Wang J. Liu J. Zhou R. Ding X. Zhang Q. Zhang C.. (2018). Zika virus infected primary microglia impairs NPCs proliferation and differentiation. Biochem. Biophys. Res. Commun. 497, 619–625. 10.1016/j.bbrc.2018.02.118 29453985

[B132] Wang Q. Lv C. Sun Y. Han X. Wang S. Mao Z.. (2018). The role of α-lipoic acid in the pathomechanism of acute ischemic stroke. Cell. Physiol. Biochem. 48, 42–53. 10.1159/000491661 29996116

[B135] Wendt S. Wogram E. Korvers L. Kettenmann H. (2016). Experimental cortical spreading depression induces NMDA receptor dependent potassium currents in microglia. J. Neurosci. 36, 6165–6174. 10.1523/JNEUROSCI.4498-15.2016 27277795 PMC6604883

[B136] Wohleb E. S. Terwilliger R. Duman C. H. Duman R. S. (2018). Stress-induced neuronal colony stimulating factor 1 provokes microglia-mediated neuronal remodeling and depressive-like behavior. Biol. Psychiatry 83, 38–49. 10.1016/j.biopsych.2017.05.026 28697890 PMC6506225

[B137] Wu Q. Cai H. Song J. Chang Q. (2017). The effects of sEH inhibitor on depression-like behavior and neurogenesis in male mice. J. Neurosci. Res. 95, 2483–2492. 10.1002/jnr.24080 28699310

[B138] Yang J. Qi F. Gu H. Zou J. Yang Y. Yuan Q.. (2016). Neonatal BCG vaccination of mice improves neurogenesis and behavior in early life. Brain Res. Bull. 120, 25–33. 10.1016/j.brainresbull.2015.10.012 26536170

[B139] Yang W. Zhou K. Zhou Y. An Y. Hu T. Lu J.. (2018). Naringin dihydrochalcone ameliorates cognitive deficits and neuropathology in APP/PS1 transgenic mice. Front. Aging Neurosci. 10:169. 10.3389/fnagi.2018.00169 29922152 PMC5996202

[B140] Yirmiya R. Rimmerman N. Reshef R. (2015). Depression as a microglial disease. Trends Neurosci. 38, 637–658. 10.1016/j.tins.2015.08.001 26442697

[B141] You Z. Luo C. Zhang W. Chen Y. He J. Zhao Q.. (2011). Pro- and anti-inflammatory cytokines expression in rat’s brain and spleen exposed to chronic mild stress: involvement in depression. Behav. Brain Res. 225, 135–141. 10.1016/j.bbr.2011.07.006 21767575

[B142] Young E. A. Altemus M. Lopez J. F. Kocsis J. H. Schatzberg A. F. DeBattista C.. (2004). HPA axis activation in major depression and response to fluoxetine: a pilot study. Psychoneuroendocrinology 29, 1198–1204. 10.1016/j.psyneuen.2004.02.002 15219644

[B143] Young J. J. Bruno D. Pomara N. (2014). A review of the relationship between proinflammatory cytokines and major depressive disorder. J. Affect. Disord. 169, 15–20. 10.1016/j.jad.2014.07.032 25128861

[B144] Yuan J. Ge H. Liu W. Zhu H. Chen Y. Zhang X.. (2017). M2 microglia promotes neurogenesis and oligodendrogenesis from neural stem/progenitor cells via the PPARγ signaling pathway. Oncotarget 8, 19855–19865. 10.18632/oncotarget.15774 28423639 PMC5386728

[B147] Zhang J. Groff R. F. Dayawansa S. (2013). Imipramine treatment increases cell proliferation following fluid percussion brain injury in rats. Neurol. Res. 35, 247–254. 10.1179/1743132813Y.0000000164 23485052

[B149] Zhang J. Q. Wu X. H. Feng Y. Xie X. F. Fan Y. H. Yan S.. (2016). Salvianolic acid B ameliorates depressive-like behaviors in chronic mild stress-treated mice: involvement of the neuroinflammatory pathway. Acta Pharmacol. Sin. 37, 1141–1153. 10.1038/aps.2016.63 27424655 PMC5022100

[B148] Zhang J. Xie X. Tang M. Zhang J. Zhang B. Zhao Q.. (2017). Salvianolic acid B promotes microglial M2-polarization and rescues neurogenesis in stress-exposed mice. Brain Behav. Immun. 66, 111–124. 10.1016/j.bbi.2017.07.012 28736034

[B146] Zhang F. Zhong R. Li S. Fu Z. Cheng C. Cai H.. (2017). Acute hypoxia induced an imbalanced M1/M2 activation of microglia through NF-kappaB signaling in Alzheimer’s disease mice and wild-type littermates. Front. Aging Neurosci. 9:282. 10.3389/fnagi.2017.00282 28890695 PMC5574879

